# ML Models Built Using Clinical Parameters and Radiomic Features Extracted from ^18^F-Choline PET/CT for the Prediction of Biochemical Recurrence after Metastasis-Directed Therapy in Patients with Oligometastatic Prostate Cancer

**DOI:** 10.3390/diagnostics14121264

**Published:** 2024-06-15

**Authors:** Luca Urso, Corrado Cittanti, Luigi Manco, Naima Ortolan, Francesca Borgia, Antonio Malorgio, Giovanni Scribano, Edoardo Mastella, Massimo Guidoboni, Antonio Stefanelli, Alessandro Turra, Mirco Bartolomei

**Affiliations:** 1 Department of Translational Medicine, University of Ferrara, 44121 Ferrara, Italy; luca.urso@unife.it (L.U.); ctc@unife.it (C.C.); rtlnma@unife.it (N.O.); francesca.borgia@unife.it (F.B.); massimo.guidoboni@ospfe.it (M.G.); 2 Nuclear Medicine Unit, Onco-Hematology Department, University Hospital of Ferrara, 44124 Ferrara, Italy; m.bartolomei@ospfe.it; 3 Medical Physics Unit, University Hospital of Ferrara, 44124 Ferrara, Italy; edoardo.mastella@ospfe.it (E.M.); a.turra@ospfe.it (A.T.); 4 U.O.C. Radiotherapy, University Hospital of Ferrara, 44124 Ferrara, Italy; a.malorgio@ospfe.it (A.M.); a.stefanelli@ospfe.it (A.S.); 5 Department of Physics and Earth Science, University of Ferrara, 44121 Ferrara, Italy; scrgnn@unife.it; 6 U.O.C. Clinical Oncology, University Hospital of Ferrara, 44124 Ferrara, Italy

**Keywords:** biochemical recurrence, prostate cancer, ^18^F-choline PET, metastasis-directed therapy (MDT), radiomics, machine learning

## Abstract

Oligometastatic patients at [^18^F]F-Fluorocholine (^18^F-choline) PET/CT may be treated with metastasis-directed therapy (MDT). The aim of this study was to combine radiomic parameters extracted from ^18^F-choline PET/CT and clinical data to build machine learning (ML) models able to predict MDT efficacy. Methods: Oligorecurrent patients (≤5 lesions) at ^18^F-choline PET/CT and treated with MDT were collected. A per-patient and per-lesion analysis was performed, using 2-year biochemical recurrence (BCR) after MDT as the standard of reference. Clinical parameters and radiomic features (RFts) extracted from ^18^F-choline PET/CT were used for training five ML Models for both CT and PET images. The performance metrics were calculated (i.e., Area Under the Curve—AUC; Classification Accuracy—CA). Results: A total of 46 metastases were selected and segmented in 29 patients. BCR after MDT occurred in 20 (69%) patients after 2 years of follow-up. In total, 73 and 33 robust RFTs were selected from CT and PET datasets, respectively. PET ML Models showed better performances than CT Models for discriminating BCR after MDT, with Stochastic Gradient Descent (SGD) being the best model (AUC = 0.95; CA = 0.90). Conclusion: ML Models built using clinical parameters and CT and PET RFts extracted via ^18^F-choline PET/CT can accurately predict BCR after MDT in oligorecurrent PCa patients. If validated externally, ML Models could improve the selection of oligorecurrent PCa patients for treatment with MDT.

## 1. Introduction

In the last few years, massive improvements have been made in the diagnostic and therapeutic management of prostate cancer (PCa). Nevertheless, biochemical recurrence (BCR) after primary treatments still occurs in nearly one-third of PCa patients treated with radical intent [1]. The early phases of PCa recurrence are often associated with oligometastatic disease, which is commonly defined as a low-volume metastatic disease with evidence of up to five metastases [2]. The treatment of this PCa setting is still highly debated [3]. Although androgen deprivation therapy (ADT) combined with androgen receptor signaling inhibitors (ARSI) is currently suggested for use in oligometastatic PCa patients by international guidelines, there is growing literature evidence supporting metastasis-directed therapy (MDT) with stereotactic body radiation therapy (SBRT) [4,5,6]. MDT may ensure the local control of treated lesions, hindering metastasis-to-metastasis spread and delaying the beginning of systemic therapies [2,7].

The availability of reliable imaging plays a crucial role in guiding accurate MDT. In this respect, the increasing use of prostate-dedicated Positron Emission Tomography (PET) has highly revolutionized the diagnostic management of PCa patients. [^18^F]F-Fluorocholine (^18^F-choline) and [^68^Ga]Ga-prostate-specific membrane antigen (68Ga-PSMA) PET/Computed Tomography (CT) enable the highly accurate detection of PCa metastases, both in patients with BR and low PSA values, allowing for accurate MDT [8,9,10]. However, the earlier detection of metastases allowed by using PET imaging may determine a stage migration that does not necessarily impacts patients’ prognoses, configuring a “Will-Rogers phenomenon”, whose relevance still needs to be clarified via well-conducted trials [11]. Moreover, PET-derived parameters predictive of successful MDT are still scarcely described in the literature.

Among the cutting-edge applications of medical imaging, radiomics is surely worthy of mention. Indeed, medical images conceal information undetectable to the human eye. In this scenario, radiomics analysis aims to extract a high amount of quantitative information hidden within medical multimodal images and correlate them with clinical and/or pathological parameters [12]. In a modern concept of personalized medicine, radiomic analysis represents a useful “free” source of information that can be used for the building of predictive models, with potentially relevant diagnostic and therapeutic implications. The need to handle large amounts of data in order to build prediction models has led to the spread of artificial intelligence (AI) algorithms. Machine learning (ML) is a branch of AI in which, based on the training dataset that is first provided, the algorithm develops its own logic for decision-making or regression problems [13].

In the field of nuclear medicine, ML Models developed using PET/CT data have already demonstrated numerous applications in multiple anatomical districts and for different purposes (i.e., for the diagnosis, staging or prediction of response to treatment) [14,15,16,17].

The aim of this study was to combine clinical data with conventional and radiomic parameters extracted from ^18^F-choline PET/CT to build ML Models able to predict the efficacy of MDT in oligorecurrent PCa patients.

## 2. Materials and Methods

### 2.1. Study Population and Data Collection

This is a retrospective monocenter study involving patients treated with MDT guided by ^18^F-choline PET/CT between 2017 and 2020 at the Sant’Anna University Hospital of Ferrara (Italy).

The inclusion criteria of this study were as follows: (a) a histologically confirmed diagnosis of PCa; (b) an age > 18 years; (c) oligorecurrent disease (up to 5 lesions), including lymph node or distant metastases at ^18^F-choline PET-CT; (d) MDT guided by ^18^F-choline PET-CT and delivered through SBRT within 3 months of the PET/CT scan; (e) 2 years of follow-up available after MDT. The exclusion criteria of this study were as follows: (a) s lack of clinical and/or follow-up data; (b) systemic therapy (including ADT or chemotherapy) ongoing at the administration of MDT.

Extracted data were analyzed both per-patient and per-lesion. The main clinical and laboratory data collected included age, International Society of Urological Pathology (ISUP) grade group, and prostate-specific antigen (PSA) serum levels, both before and after MDT. Patients were divided into a high-grade group (ISUP 4,5) and a low-grade group (ISUP 1–3).

This study was performed according to the Declaration of Helsinki, Good Clinical Practice, and local ethical regulations. The local ethical committee approved this study (CE-AVEC–registration number 2/2024/Oss/AOUFe), and patients’ written informed consent was collected.

### 2.2. MDT and Follow-Up

All patients underwent SBRT to treat all metastatic lesions. Computed Tomography (CT) scans were acquired according to our clinical protocol in supine position with 2 mm slice thickness. According to the morphological and functional imaging information, the gross tumor volume (GTV) was segmented. SBRT was delivered in 3–5 fractions using a Versa HD linear accelerator (Elekta AB, Stockholm, Sweden) equipped with a standard Agility multileaf collimator. Volumetric modulated arc therapy (VMAT) was planned with multiple flattening filter free (FFF) beams, generally two. More details about treatment planning are described in previously published papers [18]. Daily in-room 3D cone beam (CB) CT was required for pre-treatment patient position verification.

Serial PSA dosages were performed every 3 months following MDT to check its efficacy. Biochemical recurrence (BCR) was considered the main outcome of MDT, defined as the increase in serum PSA values higher than 2 ng/mL with respect to the lower PSA value reached following MDT. None of the patients included showed evidence of progression at medical imaging earlier than BCR. Patients were dichotomized as BCR0 or BCR1 according to the absence or evidence of BCR, respectively.

### 2.3. ^18^F-Choline PET/CT Acquisition Protocol and Analysis

The ^18^F-choline PET/CT images were acquired from the mid-thigh to the skull vertex 60 ± 10 min after the administration of 3 MBq/kg of ^18^F-choline, using a standard technique on a dedicated 3D PET/CT tomograph (Biograph mCT Flow; Siemens Medical Solutions, Malvern, PA, USA). All the patient’s images were acquired and processed with the same protocol on the same PET/CT tomograph, allowing us to avoid inter-scanner heterogeneity. Following non-contrast-enhanced low-dose CT (120 keV, 80 mAs, CareDose; reconstructed with a soft-tissue kernel to a slice thickness of 3 mm), emissive PET imaging was acquired in 3D mode (matrix, 200 × 200) using a continuous scan with variable speed according to the body district. Raw data were corrected for randoms, scatter, and attenuation using the co-registered non-diagnostic CT.

**Figure 1:**
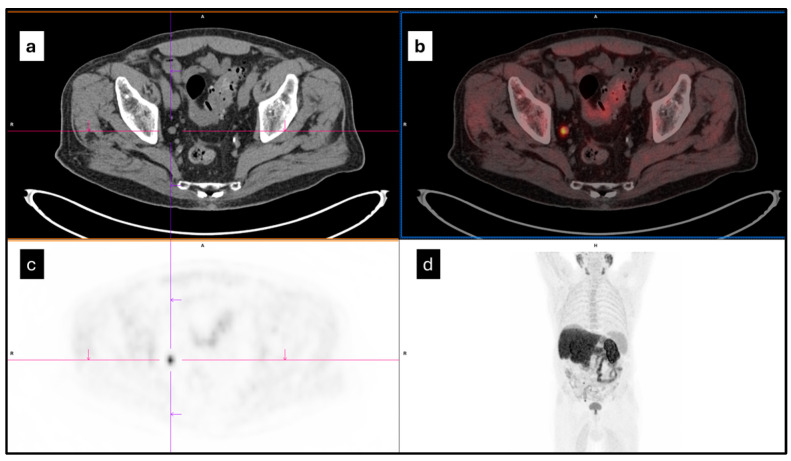
Axial images of CT (**a**), PET/CT (**b**), PET (**c**), and MIP (maximum intensity projection) (**d**) of a 81-year-old man created by ^18^F-choline PET/CT. A focal ^18^F-choline uptake (SUVmax = 15.6) was identified in the right obturator node.

The ^18^F-choline PET/CT images were critically analyzed by two experienced nuclear medicine physicians (L.U and C.C.) on a syngo.via workstation (Siemens Healthineers, Enlargen, Germany) as showed in Figure 1. Pathological findings were considered to be area(s) of focal increased radiotracer uptake outside the sites of physiological distribution. Any discrepancies were resolved by consensus. Circular manual regions of interest (ROIs) were drawn around the pathological findings and automatically converted in 3D volumes of interest (VOIs) by the software.

Metabolic and volumetric parameters extracted from ^18^F-choline PET/CT comprehended maximum and mean standardized uptake value (SUVmax and SUVmean, respectively); metabolic tumor volume (MTV), representing the tumor volume with at least 40% uptake of the SUVmax within the VOI; and total lesion choline kinase activity (TLCKa), calculated by multiplying SUVmean and MTV within the same VOI.

### 2.4. Image Segmentation and Feature Extraction

**Figure 2:**
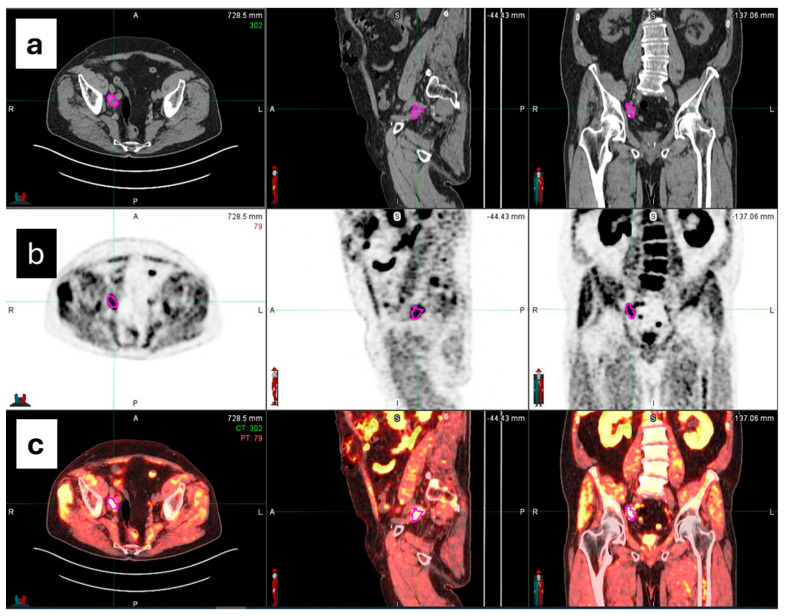
The manual segmentation of volumetric regions of interest (VOIs) on PET/CT images (**c**) around the area of focal ^18^F-choline uptake using MiM Maestro software. Each contour (pink line) was superimposed on CT (**a**) and PET (**b**) images.

VOI segmentations of PCa metastasis were manually performed on ^18^F-choline PET/CT images by two expert nuclear medicine physicians using MIM maestro version 7.3.2 (MIM Software, Inc., Cleveland, OH, USA) as reported in Figure 2. Any discrepancies were resolved by consensus.

Quantitative radiomics features were extracted from each VOI on both PET and CT images separately using the Radiomics package and 3D Slicer image-computing platform according to IBSI standardization [19].

For each VOI, 121 radiomic features (RFts) were extracted from the original images. Among those, which were, respectively, divided by classes, 14 RFts belong to the original image and mask, 14 to the shape (3D) class, 18 to first-order intensity statistics, 24 to the gray-level co-occurrence matrix (GLCM), 16 to the gray-level run length matrix (GLRLM), 16 to gray-level size zone (GLSZM), 14 to the gray-level dependence matrix (GLDM), and 5 to the neighboring gray-tone difference matrix (NGTDM). In addition, 744 textural RFts were extracted from wavelet-decomposed VOIs.

### 2.5. Statistical Analysis and Model Building

The correlation between clinical parameters, ^18^F-choline metabolic and volumetric PET/CT parameters, MDT doses, and the BCR group was investigated both per-patient and per-lesion, using the two-tailed Mann–Whitney U-type test.

A filter features selection step, performed using a hand-crafted algorithm in the Python language, was used to identify robust CT and PET RFts based on the Mann–Whitney U-type test and the Spearman rank correlation coefficient.

A *p*-value < 0.05 and Rs coefficient > 0.8 were considered significant to select the most robust and non-redundant RFts. This analysis was independently performed for both CT-based and PET-based RFt datasets. The specific results of the feature selection step are reported in the Supplementary Materials.

Selected robust RFts and clinical parameters were used to build multiple PET and CT ML Models. The whole population was split into training-test (70%) and validation (30%) datasets. The Synthetic Minority Over-Sampling Technique (SMOTE) was used to balance the sample distribution of BCR0 and BCR1 across the 2 sets, obtaining a sample size of 70 lesions, with 35 BCR0 and 35 BCR1.

Orange Data Mining, an open-source toolkit, was used to train, test, and validate five different ML algorithms, both for PET and CT: Decision Tree (Tree), Random Forest (RF), Gradient Boosting (GB), Stochastic Gradient Descent (SGD) and Support Vector Machines (SVMs). All the learners were tested via 10-Fold Cross-Validation using 70% of the main dataset. The validation step was conducted on the remaining 30% of the set-aside dataset. Performance models were evaluated in terms of AUC, Classification Accuracy (CA), Precision and Sensitivity, Positive Predictive Value (PPV), and Negative Predictive Value (NPV).

## 3. Results

### 3.1. Population Analysis

Overall, 29 oligorecurrent PCa patients with a mean age of 71 ± 7 years previously treated with radical prostatectomy (86%) or radiation therapy (RT) with radical intent alone (7%) or associated with 6–12 months of adjuvant ADT (7%) were retrieved. Evidence of oligorecurrent disease occurred after a mean period of 5 (1–8) years from primary treatment.

Overall, 46 metastases were detected through ^18^F-choline PET/CT (36 lymph nodes, 10 bone metastases), corresponding to 1.6 lesion per patient. None of the patients investigated had evidence of visceral metastases at ^18^F-choline PET/CT.

Evidence of BCR (BCR1) was considered the main outcome after MDT. In 2-year follow-up period, BCR occurred in 20 (69%) patients after a mean period of 12.8 ± 13.2. In a per-lesion analysis, 35 (76.1%) lesions belonged to the BCR1 group. Detailed characteristics of the patients included are reported in Table 1.

### 3.2. ^18^F-Choline PET/CT Parameters and PSA Analysis

All clinical parameters (per-patient analysis), metabolic and volumetric parameters (per-lesion analysis) derived from ^18^F-choline PET/CT, and MDT doses were unable to distinguish between BCR0 and BCR1 groups (Table 2 and Table 3).

### 3.3. Radiomic Analysis and Model Building

Among the 854 RFts extracted from original and filtered images, significant statistical differences between BCR0 and BCR1 groups were found for a total of 73 and 33 robust RFts for the CT and PET image datasets, respectively. The selected features belong to the first- (4) and second-order GLCMs (3) and textural wavelet-based (66) class for CT. All 33 robust PET RFts belong to the textural wavelet-based class.

The 10-Fold CV in CT Model showed an AUC > 0.80 and a CA > 0.80. Similarly, in the PET Model, an AUC > 0.95 and a CA > 0.90 were obtained.

The classification performances of the validation step are listed in Table 4. The CT Models showed good performances (AUC, CA, precision, sensitivity, PPV, and NPV were all above 0.70) using the GB and SGD algorithms, although the best performances were obtained via RF (AUC = 0.92; CA = 0.85; sensitivity = 0.85, PPV = 0.91; NPV = 0.74).

**Figure 3:**
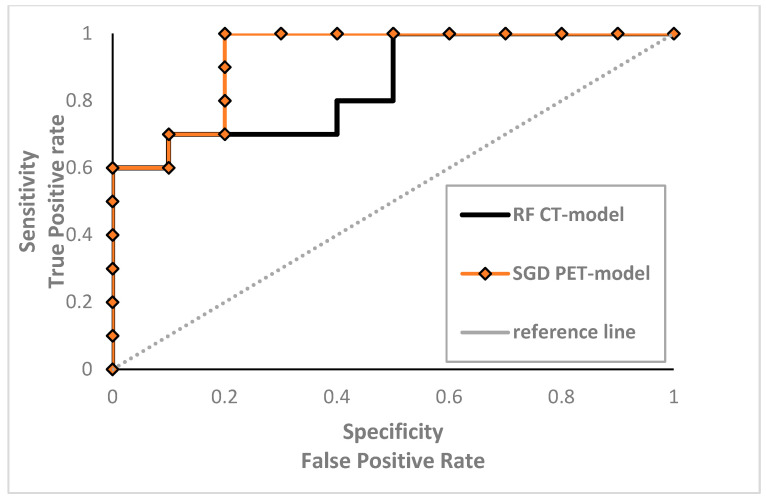
Comparison of the ROC curves for Random Forest CT Model and Stochastic Gradient Descent PET Model. RF: Random Forest; SGD: Stochastic Gradient Descent.

For the PET Model, SGD obtained the best performance (AUC = 0.95; CA = 0.90; sensitivity = 0.90; PPV = 0.94; NPV= 0.93), followed by the SVM model (AUC = 0.90; CA = 0.75; sensitivity = 0.75; PPV= 0.91; NPV = 0.63). The ROC curves of the best ML Models are graphed in Figure 3.

Predictive models trained on radiomic patterns relative to the same kinds of metastases (i.e., bone or lymph nodes) were evaluated. In the case of bone-lesions, the sample size (10) and unbalanced population (1 BCR0 vs. 9 BCR1) did not allow adequate model training in either PET or CT.

Considering lymph node metastases, 31 robust RFts were selected (3 belonging to the first-order GLCM, 3 belonging to the second-order GLCM, 25 belonging to the textural wavelet-based class) and 18 RFts were selected (wavelet class) for the CT and PET datasets, respectively.

The same five algorithms used for the whole cohort (GB, RF, SGD, SVM, Tree) were also used for the training of the PET and CT Models relative to lymph node-lesions. In 10-Fold CV, AUC < 0.75 (0.48–0.75) and AUC < 0.85 (0.40–0.85) were obtained for the PET and CT Models, respectively. Moreover, all the CAs were less than 0.70. The validation step in the CT Models achieved unsatisfactory performances (AUC 0.34–0.71 and CA < 0.70). In the PET Model, the promising results in the training step were not matched by equally good performances in the validation step (AUC 0.30–0.58 and CA < 0.68).

## 4. Discussion

In the last few years, MDT has progressively gained relevance in the treatment of oligorecurrent PCa. Indeed, evidence in support of MDT is accumulating in the literature, with papers reporting that this therapeutic strategy enables researchers to postpone systemic drug administration and improves PCa patients’ outcomes [20,21,22,23]. However, patient selection and the timing of MDT remain challenging and unsolved issues [22]. Accurate imaging modalities able to detect as many sites of recurrence as possible play a crucial role in guiding effective MDT [24]. In PCa, PET imaging is currently the most accurate imaging modality for biochemical recurrence detection [25]. Although PSMA-ligand PET currently represents the gold-standard method of examination, in particular for patients with low PSA recurrence, its introduction into daily clinical practice is relatively recent, and long-term outcome data are barely available. Therefore, the analysis of data related to other PET imaging modalities previously used for guiding MDT, including ^18^F-choline PET/CT, still represents an inestimable opportunity to understand the role of imaging in guiding MDT. In particular, we still need to identify predictive factors that can be collected from baseline imaging and concur with the prediction of the long-term outcomes of MDT in oligorecurrent PCa patients.

In our sample of oligorecurrent PCa patients treated with MDT guided via ^18^F-choline PET/CT, 2-year BCR occurred in 69% of patients (corresponding to a 2-year PFS—progression free survival—rate of 31%) after a mean period of 12.8 months from radiation therapy initiation. Our data are slightly below those reported in the literature, with 2-year PFS ranging between 35 and 44% [26,27]. In our cohort, both clinical and conventional ^18^F-choline PET/CT parameters (including SUVmax, SUVmean, MTV, and TCKa) failed to predict BCR after MDT. Our finding is consistent with the results previously published by Cysouw et al. [28], who reported that ^18^F-choline PET/CT SUV parameters and TLCKa are not significantly predictive of PFS in oligometastatic PCa.

Therefore, our attention shifted towards radiomics and AI. To the best of our knowledge, this is the first study to report a radiomic analysis of ^18^F-choline PET/CT in oligorecurrent PCa patients treated with MDT. A high number of robust RFts able to discriminate BCR0 from BCR1 groups were extracted from PET (n.33) and CT images (n.73). This finding highlights the added value of radiomic analysis in comparison to standard parameters currently applied in the daily clinical practice of PCa imaging [29]. Interestingly, all the robust RFts extracted from the PET dataset belong to the textural wavelet-based class (i.e., calculated for the filtered image/VOI). This finding reflects the fact that high- and low-frequency information extracted from PET images provides powerful informative data for use in a predictive model.

Robust RFts extracted from PET and CT images were used for training five ML Models for each dataset. We applied 10-fold CV to increase the amount of data available for training the models. Interestingly, the SGD PET Model was very accurate regarding the discrimination of the two groups, achieving very high performances (AUC = 0.95; CA = 0.90; sensitivity = 0.90; PPV = 0.94; NPV= 0.93), superior to those obtained with other PET and CT Models. The second best model was the RF CT Model (AUC = 0.92; CA = 0.85; sensitivity = 0.85, PPV = 0.91; NPV = 0.74). The validation of these two ML Models in external datasets with large sample sizes is desirable. If our data are confirmed, the potential inclusion of these ML Models in daily clinical practice could represent a relevant step towards a modern concept of personalized medicine. Indeed, an accurate tool that can predict patients unlikely to experience long-term benefits from MDT might suggest closer follow-up monitoring or even a direct switch to systemic therapies.

SGD was the best PET Model and showed better performances in comparison to RF, which was the best CT Model. We can speculate that the PET Model was more accurate because we analyzed a heterogeneous group of metastases (36 lymph nodes and 10 bone metastases). Indeed, ^18^F-choline PET imaging reflects the metabolic activity of PCa cells within a lesion, being an in vivo representation of choline metabolism [30]. Conversely, CT is a morphologic imaging method, and a metastatic lymph node is surely very different from a bone metastasis in terms of radiologic appearance. Therefore, we tried to split our population according to the site of each metastasis, analyzing separately the groups of lymph node and bone metastases. As expected, models built on bone metastases showed unsatisfactory results due to the too-small number of lesions collected and the unbalanced population (only 10% of BCR0). Considering the lymph node metastasis cohort, the results obtained using both the CT and PET Models could not pair with those of the whole cohort. The reasons are related to the low number of robust features able to discriminate between the two classes. In addition, the low number of instances available, compared to the whole cohort case, meant that we built models that showed worse performances during validation due to an overfitting problem.

The current study has some limitations. Firstly, the retrospective nature of this study and the small sample size should be mentioned as the main limitations. Finally, the patients analyzed received MDT guided by ^18^F-choline and not PSMA-ligand PET/CT because they date from a period preceding the adoption of PSMA PET in our institute. This work may represent a model that can be replicated in a future prospective study using PSMA-ligand PET/CT, which is currently indicated as the imaging method of choice for guiding MDT in oligorecurrent prostate cancer patients [31].

## 5. Conclusions

Our results highlight that radiomic analysis and ML Models can be useful for the prediction of the outcomes of MDT in oligorecurrent PCa patients. Further efforts should be made for collecting larger sample size and for an external validation of our preliminary results. Furthermore, future studies should also explore radiomic analysis and ML Models in oligorecurrent PCa patients treated with MDT guided via PSMA-ligand PET/CT, which is currently the imaging method of choice for this clinical indication.

## Figures and Tables

**Table 1:** Population characteristics.

Patients Characteristics	BCR0	BCR1	Total
Number of patients	9	20	29
AGE [years]			
Mean ± St.dev.	71 ± 6	71 ± 7	71 ± 7
Range	61–81	60–81	60–81
PSA [ng/mL]			
Mean ± St.dev.	2.63 ± 3.90	2.17 ± 2.68	2.31 ± 3.05
Range	0.01–12.06	0.24–9.00	0.01–12.06
ISUP grade			
1	0 (0%)	4 (20.0%)	4 (13.8%)
2	2 (22.2%)	5 (25.0%)	7 (24.1%)
3	3 (33.3%)	3 (15.0%)	6 (20.7%)
4	3 (33.3%)	4 (20.0%)	7 (24.2%)
5	0 (0%)	2 (10.0%)	2 (6.9%)
nd	1 (11.2%)	2 (10.0%)	3 (10.3%)
N. lesions per patient			
1	7 (78.8%)	8 (40.0%)	17 (51.7%)
2	2 (22.2%)	10 (50.0%)	10 (41.3%)
3	0%	1 (5%)	1 (3.5%)
4	0%	1 (5%)	1 (3.5%)
5	0%	0%	0%

PSA: prostate-specific antigen; ISUP: International Society of Urological Pathology.

**Table 2:** Per-patient analysis of clinical parameters.

Per-Patient Analysis	BCR0	BCR1	Total	*p*	rs
Number of patients	9	20	29		
AGE [years]				0.94	0.34
Mean ± St.dev.	71 ± 6	71 ± 7	71 ± 7		
Range	61–81	60–81	60–81		
PSA [ng/mL]				0.85	0.38
Mean ± St.dev.	2.63 ± 3.90	2.17 ± 2.68	2.31 ± 3.05		
Range	0.01–12.06	0.24–9.00	0.01–12.06		
ISUP grade				0.22	
1–3	5 (55.6%)	12 (60.0%)	17 (58.6%)		
4–5	4 (44.4%)	8 (40.0%)	12 (41.4%)		

PSA: prostate-specific antigen; ISUP: International Society of Urological Pathology.

**Table 3:** Per-lesion analysis of metabolic and volumetric ^18^F-choline PET/CT and MDT parameters.

Per-Lesion Analysis	BCR0	BCR1	Total	*p*	r_s_
Number of lesions	11 (23.9%)	35 (76.1%)	46		
SUVmax				0.09	0.09
Mean ± St.dev.	14.42 ± 10.79	9.31 ± 7.31	10.53 ± 8.43		
Range	3.6–40.4	1.7–30.0	1.7–40.4		
MTV				0.10	0.18
Mean ± St.dev.	0.95 ± 0.87	1.27 ± 1.02	1.19 ± 0.99		
Range	0.2–2.7	0.5–3.8	0.2–3.8		
TLCKA				0.54	0.53
Mean ± St.dev.	8.28 ± 7.94	8.82 ± 11.75	8.63 ± 10.89		
Range	1.7–24.3	0.2–46.4	0.2–46.4		
MDT Dose per fraction [Gy]				0.20	0.31
Mean ± St.dev.	30.91 ± 3.16	32.93 ± 3.67	32.42 ± 3.60		
Range	24–36	27–38	24–38		
Number of fractions (occurrence)				0.33	0.28
	3 (73%) 4(27%)	3 (77%) 4 (17%) 5(6%)	3 (76%) 4 (19%) 5 (5%)		
BED [Gy]				0.12	0.41
Mean ± St.dev.	126.56 ± 24.15	147.95 ± 30.65	140.95 ± 30.09		
Range	88–180	108–180	88–180		

MDT: metastasis-directed therapy; BED: biological equivalent dose; SUV standardized uptake value; MTV: metabolic tumor volume; TLCKA: total lesion choline kinase activity.

**Table 4:** Classification performances of CT and PET ML Models. Values greater than 0.7 are considered acceptable.

CT Model	AUC	CA	Precision	Sensitivity	PPV	NPV
GB	0.87	0.80	0.82	0.84	0.85	0.83
RF	0.92	0.85	0.88	0.85	0.91	0.74
SGD	0.71	0.75	0.75	0.75	0.89	0.75
SVMs	0.44	0.45	0.44	0.45	0.34	0.67
Tree	0.65	0.65	0.79	0.65	0.34	0.91
**PET Model**						
GB	0.53	0.57	0.50	0.50	0.48	0.64
RF	0.79	0.70	0.70	0.70	0.71	0.73
SGD	0.95	0.90	0.90	0.90	0.94	0.93
SVMs	0.90	0.75	0.77	0.75	0.91	0.63
Tree	0.62	0.65	0.67	0.65	0.54	0.81

GB: Gradient Boosting; RF: Random Forest; SGD: Stochastic Gradient Descent; SVMs: Support Vector Machines; Tree: Decision Tree.

## Data Availability

Data are contained within the article and Supplementary Materials.

## Supplementary Materials

The following supporting information can be downloaded via this link: https://www.mdpi.com/article/10.3390/diagnostics14121264/s1, Table S1: Robust features in CT and PET datasets.
