# Supplementary material for: ML Models Built Using Clinical Parameters and Radiomic Features Extracted from 18F-Choline PET/CT for the Prediction of Biochemical Recurrence after Metastasis-Directed Therapy in Patients with Oligometastatic Prostate Cancer
Source: Diagnostics (Basel). 2024 Jun 15;14(12):1264. doi: 10.3390/diagnostics14121264 (PMC11202947; doi:10.3390/diagnostics14121264)
Supplement: Supplementary file 1 [file diagnostics-14-01264-s001.zip › diagnostics-3038698-supplementary.pdf]

Table S1. Robust features in the CT and PET datasets.

Modality	Filter	Radiomic class	Radiomic feature	BCR1			BCR0			*p*
CT	original	firstorder	10Percentile	0.00	±	0.00	23.78	±	161.90	0.017
CT	original	firstorder	Maximum	258.77	±	303.93	579.36	±	570.79	0.010
CT	original	firstorder	Mean	34.30	±	138.73	131.22	±	244.21	0.020
CT	original	firstorder	Median	31.46	±	141.51	135.68	±	248.46	0.028
CT	original	glcm	Correlation	0.63	±	0.14	0.55	±	0.10	0.039
CT	original	glszm	SizeZoneNonUniformityNormalized	0.27	±	0.09	0.33	±	0.09	0.046
CT	original	glszm	SmallAreaEmphasis	0.51	±	0.10	0.58	±	0.09	0.045
CT	wavelet-LHL	firstorder	Skewness	0.02	±	0.37	-0.89	±	1.89	0.029
CT	wavelet-LHL	glcm	ClusterShade	-15.90	±	81.08	-47.67	±	86.47	0.014
CT	wavelet-LHH	firstorder	Maximum	55.34	±	34.61	83.18	±	53.37	0.037
CT	wavelet-LHH	ngtdm	Busyness	10.70	±	8.01	7.20	±	9.84	0.042
CT	wavelet-LHH	ngtdm	Strength	0.12	±	0.17	0.28	±	0.26	0.037
CT	wavelet-HLL	gldm	LargeDependenceLowGrayLevelEmphasis	4.58	±	3.68	2.13	±	2.38	0.039
CT	wavelet-HLL	gldm	LowGrayLevelEmphasis	0.08	±	0.05	0.04	±	0.03	0.035
CT	wavelet-HLL	glrlm	LongRunLowGrayLevelEmphasis	0.16	±	0.12	0.08	±	0.08	0.029
CT	wavelet-HLL	glrlm	LowGrayLevelRunEmphasis	0.08	±	0.05	0.04	±	0.04	0.037
CT	wavelet-HLL	glrlm	ShortRunLowGrayLevelEmphasis	0.06	±	0.04	0.03	±	0.03	0.045
CT	wavelet-HHL	firstorder	10Percentile	-19.98	±	8.57	-22.89	±	7.57	0.037
CT	wavelet-HHL	firstorder	90Percentile	19.78	±	8.25	24.62	±	9.54	0.023
CT	wavelet-HHL	firstorder	Entropy	1.49	±	0.41	1.71	±	0.38	0.019
CT	wavelet-HHL	firstorder	MeanAbsoluteDeviation	12.62	±	5.33	15.07	±	5.30	0.029
CT	wavelet-HHL	firstorder	Mean	0.03	±	0.30	0.32	±	0.48	0.023
CT	wavelet-HHL	firstorder	Range	118.73	±	77.42	173.90	±	139.16	0.047
CT	wavelet-HHL	firstorder	RootMeanSquared	16.26	±	7.43	20.07	±	7.08	0.014
CT	wavelet-HHL	firstorder	Uniformity	0.41	±	0.08	0.37	±	0.08	0.020
CT	wavelet-HHL	firstorder	Variance	317.85	±	368.01	448.06	±	344.45	0.014
CT	wavelet-HHL	glcm	ClusterProminence	15.77	±	47.43	23.27	±	30.76	0.007
CT	wavelet-HHL	glcm	ClusterTendency	1.25	±	1.43	1.63	±	1.14	0.011
CT	wavelet-HHL	glcm	Contrast	1.15	±	1.11	1.50	±	0.91	0.012
CT	wavelet-HHL	glcm	DifferenceAverage	0.72	±	0.27	0.84	±	0.25	0.020
CT	wavelet-HHL	glcm	DifferenceEntropy	1.37	±	0.31	1.54	±	0.28	0.015
CT	wavelet-HHL	glcm	DifferenceVariance	0.54	±	0.49	0.71	±	0.42	0.012
CT	wavelet-HHL	glcm	Id	0.69	±	0.06	0.66	±	0.06	0.025
CT	wavelet-HHL	glcm	Idm	0.68	±	0.07	0.64	±	0.07	0.023
CT	wavelet-HHL	glcm	JointEnergy	0.18	±	0.06	0.15	±	0.05	0.027
CT	wavelet-HHL	glcm	JointEntropy	2.89	±	0.78	3.28	±	0.70	0.020
CT	wavelet-HHL	glcm	SumEntropy	1.96	±	0.43	2.18	±	0.40	0.018
CT	wavelet-HHL	glcm	SumSquares	0.59	±	0.61	0.82	±	0.57	0.009
CT	wavelet-HHL	gldm	GrayLevelVariance	0.60	±	0.59	0.80	±	0.54	0.012
CT	wavelet-HHL	gldm	HighGrayLevelEmphasis	13.14	±	12.37	29.93	±	51.62	0.020
CT	wavelet-HHL	gldm	SmallDependenceHighGrayLevelEmphasis	1.04	±	1.37	2.26	±	3.19	0.039
CT	wavelet-HHL	glrlm	GrayLevelNonUniformityNormalized	0.39	±	0.08	0.34	±	0.07	0.019
CT	wavelet-HHL	glrlm	GrayLevelVariance	0.68	±	0.65	0.93	±	0.61	0.012
CT	wavelet-HHL	glrlm	HighGrayLevelRunEmphasis	13.19	±	12.39	30.06	±	51.69	0.013
CT	wavelet-HHL	glrlm	ShortRunHighGrayLevelEmphasis	10.35	±	10.34	22.93	±	37.53	0.035
CT	wavelet-HHL	glszm	HighGrayLevelZoneEmphasis	14.87	±	13.53	32.82	±	53.20	0.027
CT	wavelet-HHL	glszm	LargeAreaLowGrayLevelEmphasis	1597.21	±	3236.70	456.34	±	687.25	0.019
CT	wavelet-HHL	glszm	ZonePercentage	0.05	±	0.02	0.08	±	0.04	0.031
CT	wavelet-HHL	ngtdm	Busyness	13.40	±	19.65	6.28	±	6.78	0.047
CT	wavelet-HHL	ngtdm	Strength	0.11	±	0.14	0.26	±	0.26	0.047
CT	wavelet-HHH	firstorder	90Percentile	9.17	±	2.57	11.44	±	4.07	0.023
CT	wavelet-HHH	firstorder	InterquartileRange	9.44	±	2.48	11.85	±	4.22	0.033
CT	wavelet-HHH	firstorder	MeanAbsoluteDeviation	5.84	±	1.71	7.18	±	2.62	0.033
CT	wavelet-HHH	firstorder	Minimum	-29.54	±	28.47	-36.66	±	19.92	0.022
CT	wavelet-HHH	firstorder	RobustMeanAbsoluteDeviation	3.97	±	1.03	4.92	±	1.78	0.042
CT	wavelet-HHH	firstorder	RootMeanSquared	7.57	±	2.74	9.21	±	3.33	0.022
CT	wavelet-HHH	firstorder	Variance	64.57	±	55.88	94.82	±	75.58	0.022
CT	wavelet-HHH	glcm	Contrast	0.56	±	0.14	0.60	±	0.16	0.039
CT	wavelet-HHH	glcm	DifferenceAverage	0.53	±	0.04	0.55	±	0.07	0.031
CT	wavelet-HHH	glcm	Id	0.74	±	0.01	0.73	±	0.02	0.037
CT	wavelet-HHH	glcm	Idm	0.74	±	0.01	0.73	±	0.03	0.033
CT	wavelet-HHH	glcm	Imc1	-0.01	±	0.02	-0.02	±	0.01	0.014

CT	wavelet-HHH	glcm	Imc2	0.14	±	0.08	0.17	±	0.06	0.014
CT	wavelet-HHH	glcm	JointAverage	1.98	±	1.28	2.32	±	1.08	0.045
CT	wavelet-HHH	glcm	MCC	0.13	±	0.12	0.17	±	0.11	0.020
CT	wavelet-HHH	gldm	HighGrayLevelEmphasis	5.79	±	12.05	6.75	±	6.06	0.039
CT	wavelet-HHH	glszm	GrayLevelNonUniformityNormalized	0.45	±	0.09	0.38	±	0.12	0.049
CT	wavelet-HHH	ngtdm	Busyness	93.56	±	68.48	71.08	±	117.11	0.035
CT	wavelet-HHH	ngtdm	Strength	0.03	±	0.10	0.05	±	0.05	0.037
CT	wavelet-LLL	firstorder	10Percentile	-158.23	±	227.92	90.67	±	478.14	0.015
CT	wavelet-LLL	firstorder	Maximum	677.83	±	803.43	1367.08	±	1356.37	0.035
CT	wavelet-LLL	firstorder	Mean	96.02	±	392.33	368.67	±	688.72	0.023
CT	wavelet-LLL	firstorder	Median	89.14	±	401.59	379.27	±	703.47	0.033
PET	wavelet-LLH	firstorder	MeanAbsoluteDeviation	1.34	±	2.31	1.83	±	1.60	0.039
PET	wavelet-LHL	firstorder	RootMeanSquared	1.01	±	0.69	3.72	±	3.30	0.035
PET	wavelet-LHH	firstorder	90Percentile	2.18	±	2.26	1.17	±	1.57	0.025
PET	wavelet-LHH	firstorder	InterquartileRange	0.48	±	0.45	0.76	±	0.69	0.019
PET	wavelet-LHH	firstorder	MeanAbsoluteDeviation	0.41	±	0.33	0.58	±	0.58	0.037
PET	wavelet-LHH	firstorder	RootMeanSquared	0.30	±	0.27	0.78	±	0.87	0.045
PET	wavelet-LHH	firstorder	Variance	0.40	±	0.38	1.26	±	3.02	0.042
PET	wavelet-HLL	firstorder	Median	0.30	±	0.72	0.93	±	1.25	0.037
PET	wavelet-HLH	glcm	Contrast	0.26	±	1.04	0.41	±	0.15	0.016
PET	wavelet-HLH	glcm	DifferenceAverage	0.51	±	0.08	0.41	±	0.15	0.016
PET	wavelet-HLH	glcm	Id	0.51	±	0.08	0.80	±	0.08	0.016
PET	wavelet-HLH	glcm	Idm	0.75	±	0.04	0.80	±	0.08	0.016
PET	wavelet-HLH	glcm	Idmn	0.75	±	0.04	0.92	±	0.03	0.016
PET	wavelet-HLH	glcm	Idn	0.90	±	0.02	0.86	±	0.05	0.016
PET	wavelet-HLH	glcm	InverseVariance	0.83	±	0.03	0.41	±	0.15	0.016
PET	wavelet-HLH	ngtdm	Complexity	0.51	±	0.08	0.39	±	0.15	0.020
PET	wavelet-HLH	ngtdm	Contrast	0.48	±	0.08	0.09	±	0.04	0.022
PET	wavelet-HHL	firstorder	MeanAbsoluteDeviation	0.12	±	0.02	1.13	±	1.26	0.027
PET	wavelet-HHL	firstorder	RootMeanSquared	0.60	±	0.63	1.55	±	1.92	0.033
PET	wavelet-HHL	firstorder	Variance	0.81	±	1.01	5.73	±	15.33	0.035
PET	wavelet-HHH	firstorder	Median	1.60	±	5.71	0.04	±	0.09	0.014
PET	wavelet-HHH	firstorder	Skewness	0.00	±	0.03	-0.73	±	0.94	0.027
PET	wavelet-HHH	glcm	JointAverage	0.01	±	0.63	1.56	±	0.07	0.027
PET	wavelet-LLL	glcm	ClusterShade	1.49	±	0.11	0.06	±	0.08	0.026
PET	wavelet-LLL	glcm	Correlation	-0.01	±	0.09	0.53	±	0.51	0.033
PET	wavelet-LLL	glcm	MCC	0.82	±	0.33	0.57	±	0.42	0.028
PET	wavelet-LLL	glszm	HighGrayLevelZoneEmphasis	0.82	±	0.31	2.06	±	1.19	0.045
PET	wavelet-LLL	glszm	LowGrayLevelZoneEmphasis	1.40	±	0.81	0.77	±	0.23	0.045
PET	wavelet-LLL	glszm	SizeZoneNonUniformityNormalized	0.91	±	0.16	0.70	±	0.30	0.037
PET	wavelet-LLL	glszm	SmallAreaEmphasis	0.88	±	0.24	0.04	±	0.04	0.019
PET	wavelet-LLL	glszm	SmallAreaHighGrayLevelEmphasis	0.02	±	0.06	0.14	±	0.22	0.008
PET	wavelet-LLL	glszm	ZoneVariance	0.02	±	0.06	201.43	±	499.23	0.038
PET	wavelet-LLL	ngtdm	Strength	39.50	±	116.25	0.16	±	0.20	0.036
